# Imputation of missing values of tumour stage in population-based cancer registration

**DOI:** 10.1186/1471-2288-11-129

**Published:** 2011-09-19

**Authors:** Nora Eisemann, Annika Waldmann, Alexander Katalinic

**Affiliations:** 1 Institute of Cancer Epidemiology, University Luebeck, Ratzeburger Allee 160 (Haus 50), 23562 Luebeck, Germany; 2 Institute of Clinical Epidemiology, University hospital Schleswig-Holstein, Campus Luebeck, Ratzeburger Allee 160 (Haus 50), 23562 Luebeck, Germany

## Abstract

**Background:**

Missing data on tumour stage information is a common problem in population-based cancer registries. Statistical analyses on the level of tumour stage may be biased, if no adequate method for handling of missing data is applied. In order to determine a useful way to treat missing data on tumour stage, we examined different imputation models for multiple imputation with chained equations for analysing the stage-specific numbers of cases of malignant melanoma and female breast cancer.

**Methods:**

This analysis was based on the malignant melanoma data set and the female breast cancer data set of the cancer registry Schleswig-Holstein, Germany. The cases with complete tumour stage information were extracted and their stage information partly removed according to a MAR missingness-pattern, resulting in five simulated data sets for each cancer entity. The missing tumour stage values were then treated with multiple imputation with chained equations, using polytomous regression, predictive mean matching, random forests and proportional sampling as imputation models. The estimated tumour stages, stage-specific numbers of cases and survival curves after multiple imputation were compared to the observed ones.

**Results:**

The amount of missing values for malignant melanoma was too high to estimate a reasonable number of cases for each UICC stage. However, multiple imputation of missing stage values led to stage-specific numbers of cases of T-stage for malignant melanoma as well as T- and UICC-stage for breast cancer close to the observed numbers of cases. The observed tumour stages on the individual level, the stage-specific numbers of cases and the observed survival curves were best met with polytomous regression or predictive mean matching but not with random forest or proportional sampling as imputation models.

**Conclusions:**

This limited simulation study indicates that multiple imputation with chained equations is an appropriate technique for dealing with missing information on tumour stage in population-based cancer registries, if the amount of unstaged cases is on a reasonable level.

## Background

An important task of population-based cancer registration is to assess the effectiveness of early detection programmes such as mammography screening. Time trend analysis of cancer incidence is an important indicator in such an evaluation and is often conducted. Time trend analysis of tumour stage-specific incidence is more appropriate, however less frequently applied [1-3]. A reduction in incidence of tumours with a poor prognosis might indicate a future reduction in mortality. Complete stage information is crucial for such analyses. Missing values for stage information might bias such a stage-specific analysis, especially when the missingness-pattern changes over time. A cancer registry may have a very complete case registration, yet still have missing information in important parameters, such as tumour size or lymph node status, remains an almost common phenomenon in population-based cancer registration. The percentage of unknown stages can vary considerably between different cancer entities or cancer registries. Concerning melanoma and breast cancer, the federal cancer registries in Germany report the following percentages of unknown T-stage (tumour size according to TNM staging system [4]) between 10-20% [5,6] and of unknown UICC-stage [7] between 20-40% [8,9]. There are several reasons for this, one being that tumour stage is often not known at the time of diagnosis and therefore, if the case is reported to the registry without additional notification, e.g. from the physician or from the pathologists, stage information is lost. Further, some cancer cases are only reported by a pathologist. These notifications - in general - do not provide any information on lymph node status or metastasis.

Concerning statistical analyses on the level of tumour stages, three more or less common approaches for dealing with missing stage data can be found: 1. ad-hoc missing data methods, such as omitting all cases with missing information [10-12] or analysing them as a separate group [13], 2. distributing all cases with unknown stage proportionally to the known stages [14] and 3. using multiple imputation [15,16].

The first approach is widely known to produce biased results [17,18].

The second approach yields valid population-based analyses on tumour stage-specific incidence if the precondition of equal tumour stage distributions among the cases with unknown T-stage and the cases with known T-stage is met. If this is not the case, the results will be biased. Additionally, analysis on the individual level is not possible with this method. Therefore, the missing values in tumour stage should be handled with an appropriate statistical method (such as approach 3) before calculating the stage-specific incidence rates to reduce the expected bias.

The following limited simulation analysis is aimed at determining a feasible method for imputation of missing stage information in empirical cancer registry data sets. We used cancer registry data for female breast cancer (a tumour site with only few missing values) and data for malignant melanoma (a tumour site with a high proportion of missing data). The cases with complete stage information were used to derive data sets with simulated missing stage information. We then analysed the individual stage estimations, the stage-specific numbers of cases and the stage-specific survival curves after treatment with different variants of multiple imputation.

## Methods

### Databases

Malignant melanoma data (men and women, ICD-10 C43 excl. sarcomas) and breast cancer data (women; ICD-10 C50) gathered by the cancer registry Schleswig-Holstein in Germany between 2000 and 2008 was used for the following analysis. All DCO (death certificate only) cases were excluded from the analysis.

The cancer registry records data on tumour size (T-stage), involvement of lymph nodes (N-stage) and metastases (M-stage) according to the TNM-classification (see additional file 1: TNM-definition for breast cancer and malignant melanoma) [4,19]. TNM-stages can be combined to one prognostic classifier, using the UICC-classification (see additional file 2: UICC-definition) [7]. The T-classification as well as the UICC-classification consists of four main categories, with stage I having a good survival prognosis and stage IV a poor prognosis.

### Imputation of missing stage information

Our analysis consists of six main steps:

1. Selection of variables

2. Simulation of five breast cancer data sets and five malignant melanoma data sets

3. Specification of the imputation models

4. Creation of ten complete data sets out of each simulated data set using multiple imputation

5. Statistical analysis and model evaluation

6. Sensitivity analysis for malignant melanoma

#### 1. Selection of variables

Available clinically relevant variables, potentially related to stage information, were selected: sex, age at diagnosis, morphology, topography, grading, operation (yes/no), radiation therapy (yes/no), chemotherapy (yes/no), hormone therapy (yes/no), survival time and censoring. Additionally, each of the T-stage, N-stage and M-stage is associated with the two others, accordingly.

In order to take minor changes in the classification system over time and other possible temporal changes into account, year of diagnosis was also included into the analysis. As the breast cancer analysis excludes men, the variable sex was removed. No malignant melanoma patient received hormone therapy, so this variable was omitted for this data set.

Most categories in the variable morphology of the tumour had small entries. Thus, only categories affecting at least 1% of all patients were used. The other categories were pooled - with the not otherwise specified (NOS) - into one category. Topography of the tumour was treated analogously.

Most predictor variables have missing values (Table 1). This will be addressed by the multiple imputation method.

**Table 1:** Description of the observed and the simulated data sets for breast cancer and malignant melanoma patients

		Breast cancer data set		Malignant melanoma data set	
		**Observed**	Simulated*	**Observed**	Simulated*

Number of cases		21,428	17,162	5,520	1,685

Sex (in %)	Female	100	100	45.8	45.1
	Male	0	0	54.2	54.9

Age	Median (1^st ^and 3^rd ^Quartile)	62.0 (53.0; 71.0)	61.0 (52.0; 69.0)	61.0 (45.0; 70.2)	59.0 (43.0; 68.0)

T-stage (in %)	1	47.3	49.2	36.9	36.9
	2	34.0	34.3	11.8	12.4
	3	5.5	5.1	8.0	7.6
	4	7.3	5.7	4.7	4.4
	Unknown	6.0	5.7	38.6	38.7

N-stage (in %)	0	53.4	55.1	29.5	32.5
	1	25.4	25.2	1.7	1.4
	2	6.1	5.9	0.6	0.4
	3	3.7	3.5	0.2	0.1
	Unknown	11.4	10.3	67.9	65.6

M-stage (in %)	0	77.9	80.8	31.0	33.5
	1	5.6	3.7	2.2	0.7
	Unknown	16.5	15.4	66.8	65.8

UICC-stage (in %)	I	32.0	29.7	23.9	7.4
	II	33.2	30.0	3.3	0.7
	III	11.4	10.4	2.8	0.8
	IV	5.6	3.7	0.5	0.7
	Unknown	17.8	26.1	69.5	90.4

Survival time (days)	Median (1^st ^and 3^rd ^Quartile)	1279 (549; 2161)	1279 (580; 2130)	1552 (700; 2253)	1765 (975; 2557)

Censoring (in %)	Censored	84.6	88.5	88.1	90.5

Year of diagnosis	2000	10.2	9.7	10.8	15.1
	2001	10.7	10.2	11.5	14.4
	2002	11.1	10.6	10.0	10.9
	2003	10.8	10.9	14.3	12.2
	2004	10.7	10.7	12.8	14.6
	2005	10.7	10.9	10.7	8.9
	2006	11.5	11.6	10.0	10.8
	2007	11.6	12.1	9.7	5.0
	2008	12.7	13.4	10.3	8.1

Grading (in %)	1	10.6	10.6	3.4	5.7
	2	54.2	53.7	0.3	0.2
	3	30.0	30.1	0.2	0.2
	4	0.2	0.1	< 0.1	0.1
	Unknown	5.0	5.5	96.1	93.8

Radiotherapy (in %)	Yes	66.4	80.2	1.4	0.5
	no	17.8	15.3	52.2	64.5
	Unknown	15.8	4.5	46.4	35.0

Chemotherapy (in %)	Yes	46.1	47.9	1.8	1.4
	No	37.4	36.7	51.8	63.9
	Unknown	16.6	15.5	46.5	34.8

Hormone therapy (in %)	Yes	60.6	62.3	0.0	0.0
	No	19.1	18.1		
	Unknown	20.3	19.6		

Morphology (in %)	Infiltrating duct carcinoma	69.0	70.6		
	Lobular carcinoma	12.3	11.9		
	Infiltrating duct and lobular carcinoma	7.4	7.9		
	Nodular melanoma			12.4	13.6
	Lentigo maligna melanoma			5.3	5.1
	Superficial spreading melanoma			41.4	48.4
	Others and NOS	10.5	9.6	41.0	32.8

Topography (in %)	Central portion of breast	5.3	5.1		
	Upper-inner quadrant of breast	9.3	9.6		
	Lower-inner quadrant of breast	4.6	4.7		
	Upper-outer quadrant of breast	35.7	36.3		
	Lower-outer quadrant of breast	6.3	6.3		
	Axillary tail of breast	0.2	0.1		
	Overlapping lesion of breast	8.9	8.2		
	Trunc			32.2	35.9
	Extremity			46.7	50.1
	Head/Neck			13.5	11.3
	NOS	29.9	29.7	7.6	2.7

* For T-, N-, M- and UICC-stage the mean frequencies of the five simulated data sets are provided. The other variables distributions are identical in all five data sets.

#### *2. Simulation of a breast cancer data set and a malignant melanoma data set*

Conducting the multiple imputation methods on a simulated data set enables us to judge the quality of the results and to make a significant comparison between the different methods, because the true results are known. Although a proper simulation study [20] could give higher evidence, we decided to restrict the analysis to a very small simulation study with two scenarios (female breast cancer and malignant melanoma), one data generation process (described below) and five simulated data sets each, as this seemed to be a sufficient approach in recognising the possible methods and determining which of these produce acceptable results.

It was important to retain the dependencies between the variables as close as possible. Rather than simulating the data set by assuming a multivariate normal distribution of the - if necessary, transformed - variables, which would involve a certain degree of abstraction, we generated a data set using the original data set itself as the data basis: The observed female breast cancer data set *D *had approximately 21,500 cases of which 80% had no missing value in any of the variables T-, N- or M-stage. These cases were used as the observed values for the simulated data set *S*. We assumed that the missingness-pattern of stage depends mostly on age at diagnosis, survival time, censoring and the interaction between survival time and censoring. These variables were complete in *D*. A logistic regression model was fitted for each variable with missing values, with age at diagnosis, survival time, censoring and the interaction between survival time and censoring as independent variables. These models could now predict the probability of any value in *S *to be missing. Every value in *S *was deleted randomly, depending on its individual missingness-probability; a value with a high probability of missingness was therefore more probable to be deleted, but did not necessarily have to be deleted. The resulting data set was the simulated data set *S*.

The generation of missing values depended on a random starting value. Changing the random starting value would have produced a different simulated data set, which might have resulted in different conclusions about the imputation methods. To avoid such biases, we simulated a total of five data sets and obtained 50 completed data sets for each cancer entity and each variant of multiple imputation. The missingness-pattern was independent among the five data sets, but all imputation methods were conducted on the same five data sets. We used the default random number generator of R, "Mersenne-Twister", with five different starting seeds.

The observed malignant melanoma data set consisted of about 5,500 cases, of which 30% had complete T-, N- and M-stage information. The simulated data set was generated in the same way as described above.

#### 3. Specification of the imputation models

Multiple imputation with chained equations [21] was used. Four scenarios with different imputation models were compared:

(1) Polytomous logistic regression is applicable for categorial data and may be used for the T- and N-stages. However, there were only two M-stages, hence the polytomous logistic regression reduced to dichotomous logistic regression. The imputations of the missing values in the following four predictor variables morphology, radiation therapy, chemotherapy and hormone therapy were randomly sampled from the observed values.

(2) Predictive mean matching is a linear regression, in which the predicted value is substituted for the closest observed value. In our case, this yielded a value of 1, 2, 3 or 4 for T and of 0, 1, 2 or 3 for N. This method is valid for data on an ordinal scale. As for M-stage, dichotomous logistic regression was used as in scenario 1. The missing values in the predictor variables morphology, radiation therapy, chemotherapy and hormone therapy were treated as in scenario 1.

(3) The third scenario consisted of random forests [22] for T, N and M. Modern machine learning techniques are often superior to classical regression models if the modelling is complex, for example if interactions and nonlinear relations are involved [23]. The imputation models based on logistic regression and predictive mean matching included the interaction between survival time and censoring in their set of predictor variables, because a short survival time must be interpreted differently for a deceased person than for someone still alive, having only a short follow-up time. No interaction term was needed in the random forest because this method can internally model flexible interactions. The missing values in morphology, radiation therapy, chemotherapy and hormone therapy were treated as in scenario 1.

(4) The customary approach was to sample the missing tumour stage values from the observed stages, yielding a proportional distribution. To make the results comparable to the results from approach (1) to (3), multiple imputation was used rather than single imputation.

One assumption of multiple imputation is that the missing values are missing at random (MAR), e.g. the absence of a particular item is only dependent on other observable variables and not on unobservable parameters, nor the value of the item itself [23]. We included 13 predictors in the imputation models, which made the MAR assumption more plausible.

#### 4. Creation of ten complete data sets out of each simulated data sets using multiple imputation

Ten completed data sets were generated for each simulated data set for both cancer entities, using the four imputation scenarios introduced above, which is usually sufficient [24]. Gibbs sampling with ten iterations was used to ensure model convergence [25].

#### 5. Statistical analysis and model evaluation

The basic quality of the imputations was measured by the concordance of the stage predictions with their observed values and the extent of dislocation.

We then calculated T- and UICC-stage-specific numbers of cases based on the ten completed simulated data sets and compared them to the observed stage-specific numbers of cases. The numbers of cases and their standard deviations were calculated according to the rules for combining complete-data inferences [26]. The mean absolute deviation (MAD) aggregated the information on differences between the predicted and the observed stage distributions for comparison of the different methods.

Finally, we plotted Kaplan-Meier survival curves. As T- and UICC-stages are prognosis groups, the observed and the predicted stage-specific survival curves should be similar. The differences were examined with log-rank tests for each stage. The log-rank test statistics of all imputed data sets were summed up to provide a measure for the total difference and to indicate the best imputation model.

#### 6. Sensitivity analyses for malignant melanoma

Although ten imputations should generally suffice for data with a modest amount of 10-30% missingness, the malignant melanoma with 39% missing T-stages (38% in the simulated data set) and 70% missing UICC-stages (91% in the simulated data set) may require more imputations. It must be kept in mind, that a UICC-stage can already be missing if only one of the three stages (T, N, M) is missing. Although a percentage of 91% of the UICC-stages was missing, 'only' 56% of the values needed for the calculation of the UICC-stage were missing.

We repeated the analysis for malignant melanoma with 25 imputations and 50, rather than ten, iterations.

### Software

All statistical analyses were done in R 2.11.1 [27] using the packages mice [21], survival [28] and randomForest [29].

### Descriptive statistics

The percentages in the individual variable categories are given for the description of the data. The median and the first and third quartiles are shown for age and survival time.

## Results

### Missing information

There were 21,428 incident cases of female breast cancer in Schleswig-Holstein between 2000 and 2008. Six percent of the cases had no information on the T-stage, 11% had missing values in the N-stage and 16% in the M-stage. Only 17,162 (80%) cases had valid information on all three parameters.

In the same time period 5,520 cases of malignant melanoma were registered in Schleswig-Holstein. The percentage of missing values was higher than in breast cancer: 39% in T, 68% in N and 67% in M. The stage information was complete for 1,685 cases (30%). Table 1 shows the most important variables and their distributions including the number of missing values.

### Simulated data sets

The simulated data sets based on the cases with complete T-, N- and M-stage were similar to the original data sets for the relevant variables (Table 1). The only exceptions were a higher percentage of missing values for UICC-stage (27% versus 18% for breast cancer, 91% versus 70% for malignant melanoma) and a longer median survival time for malignant melanoma (1765 versus 1552 days).

The values of the cases with complete T-, N- and M-stage in the original data sets are referred to as "observed values".

### Accuracy of the imputations on individual level

Table 2 shows the concordance rate of imputed and observed T- and UICC-stages. Polytomous regression and predictive mean matching always yielded the highest concordance rate: approximately 48% of all imputations matched the observed T-stage value and approximately 80% of all imputation matched the observed UICC-stage for both cancer entities. These concordance rates were higher than can be achieved by chance, as the lower concordance rates rendered by proportional sampling indicate.

**Table 2:** Concordance rates of imputed with observed T- and UICC-stages for breast cancer and malignant melanoma

	Breast cancer				Malignant melanoma			
	**PR (in %)**	**PMM (in %)**	**RF (in %)**	**Prop (in %)**	**PR (in %)**	**PMM (in %)**	**RF (in %)**	**Prop (in %)**

T-stage								

Concordance	48.7	48.0	31.4	39.4	47.7	47.2	40.6	42.5

Dislocation by 1 stage	37.8	38.4	55.4	41.5	32.0	32.3	30.8	31.0

Dislocation by 2 stages	10.1	10.3	8.2	11.4	15.4	15.5	19.9	18.1

Dislocation by 3 stages	3.4	3.4	5.0	7.7	4.9	5.0	8.7	8.4

								

UICC-stage								

Concordance	79.5	79.1	58.8	74.2	80.6	80.8	77.9	79.5

Dislocation by 1 stage	17.3	17.6	25.0	19.9	11.1	10.8	13.3	11.5

Dislocation by 2 stages	2.9	2.9	14.2	4.8	6.2	5.7	8.1	7.4

Dislocation by 3 stages	0.3	0.3	1.9	1.1	2.1	2.7	0.6	1.6

PR Polytomous regression

PMM Predictive mean matching

RF Random forests

Prop Proportional sampling

Dislocations by three stages, i.e. a T1 (UICC I) imputed as T4 (UICC IV) or vice versa, occurred in less than 5% of all imputations for polytomous regression and predictive mean matching.

### Estimations of the stage-specific numbers of cases

Table 3 displays the observed and the predicted case numbers for T- and UICC-stage after multiple imputation with the different scenarios. For example, there were 8,909 breast cancer cases in T1-stage in the observed data set. After multiple imputation of missing values in the simulated data set, a number of 8,903.2 was predicted by the polytomous regression approach.

**Table 3:** Observed and with different multiple imputation methods predicted T- and UICC-stage-specific numbers of cases for breast cancer and malignant melanoma

		Breast cancer					Malignant melanoma				
		**Observed**	**PR**	**PMM**	**RF**	**Prop**	**Observed**	**PR**	**PMM**	**RF**	**Prop**

T-stage											

1	N	8,909.0	8,903.2	8,903.5	8,788.2	8,950.5	1,017.0	1,009.8	1,014.5	962.3	1,013.7

	*SD*	*94.4*	*96.5*	*96.5*	*108.6*	*97.6*	*31.9*	*37.4*	*38.2*	*207.2*	*37.3*

2	N	6,235.0	6,236.7	6,239.5	6,394.9	6,228.6	338.0	341.8	340.2	332.7	341.2

	*SD*	*79.0*	*82.6*	*83.0*	*83.3*	*84.1*	*18.4*	*26.8*	*29.9*	*116.8*	*25.7*

3	N	944.0	944.5	946.7	944.9	936.4	214.0	212.1	209	242.1	209.0

	*SD*	*30.7*	*33.0*	*33.3*	*33.8*	*32.7*	*14.6*	*18.7*	*19.2*	*129.6*	*17.7*

4	N	1,074.0	1,077.6	1,072.3	1,034.1	1,046.5	116.0	121.3	121.3	147.9	121.1

	*SD*	*32.8*	*36.3*	*35.9*	*57.3*	*35.3*	*10.8*	*13.6*	*15.5*	*121.8*	*16.6*

MAD			59.8	57.8	341.9	104.1		48.4	55.4	409.2	49.2

	*SD*		*23.3*	*30.1*	*97.5*	*30.4*		*23.5*	*26.1*	*242.6*	*20.3*

UICC-stage											

I	N	6,859.0	6,865.7	6,856.7	6,096.1	6,642	1,321.0	1,269.8	1,276.8	1,213.7	1,267.8

	*SD*	*82.8*	*85.4*	*85.4*	*261.0*	*85.5*	*36.3*	*39.5*	*41.1*	*222.0*	*41.7*

II	N	7,123.0	7,119.5	7,133.9	6,891.8	7,295.9	216.0	204.4	208.4	271.1	238.6

	*SD*	*84.4*	*87.5*	*87.4*	*425.7*	*88.8*	*14.7*	*24.7*	*24.8*	*143.1*	*21.5*

III	N	2,371.0	2,361.7	2,351.9	3,206.1	2,462.6	122.0	149.9	124.5	173.4	143.6

	*SD*	*48.7*	*53.2*	*53.0*	*381.6*	*58.4*	*11.0*	*21.2*	*22.9*	*199.8*	*17.5*

IV	N	809.0	815.1	819.5	968.0	761.5	26.0	60.9	75.2	26.9	35.0

	*SD*	*28.4*	*31.3*	*31.6*	*593.6*	*32.2*	*5.1*	*14.8*	*18.4*	*8.6*	*9.5*

MAD			68.5	72.7	2182.4	528.8		133.9	126.9	344.4	107.5

	*SD*		*25.8*	*30.9*	*988.5*	*47.2*		*28.1*	*35.6*	*411.8*	*41.5*

N Number of cases

SD Standard deviation

PR Polytomous regression

PMM Predictive mean matching

RF Random forests

Prop Proportional sampling

MAD Mean absolute difference between the predicted number of caces in all 50 imputations and the observed number of cases.

Polytomous regression and predictive mean matching for multiple imputation of missing T-stage had the smallest deviations from the observed case numbers for breast cancer. The best results for malignant melanoma were again achieved by these approaches and also by proportional sampling. Although the percentage of missing values was substantially higher for malignant melanoma, the results for T-stage were comparable for both breast cancer and melanoma.

Polytomous regression and predictive mean matching were also showing similar results for UICC-stage, however the proportional approach was even closer to the observed stage-specific numbers of cases for malignant melanoma.

The random forest scenario was always less accurate than the estimations by the other scenarios and had the largest standard deviations.

### Survival curve estimations

**Figure 1:**
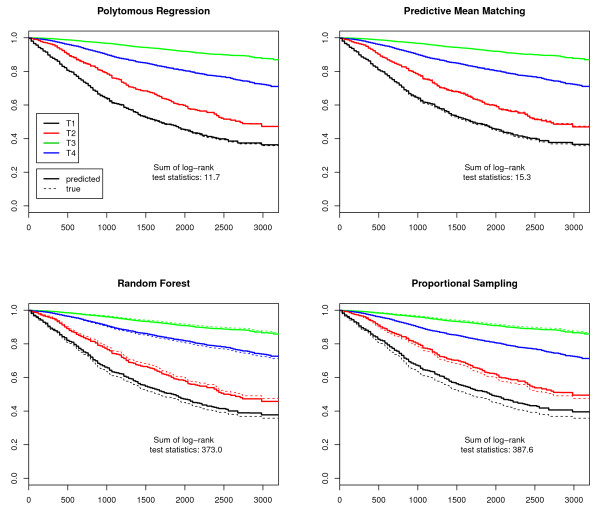
**T-stage-specific survival curves for female breast cancer**. The predicted survival curves are based on the 50 completed data sets.

**Figure 2:**
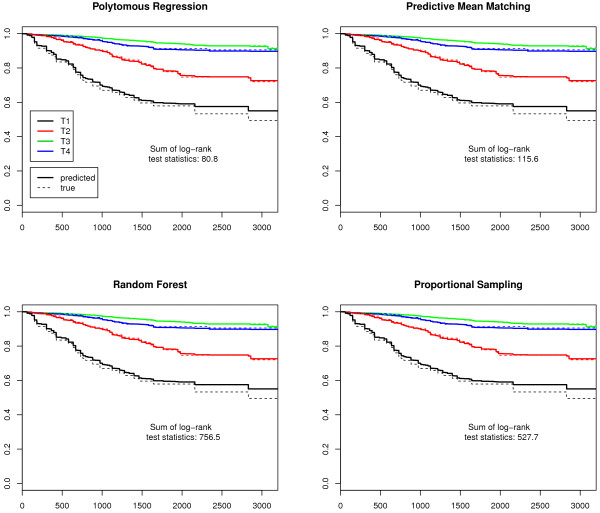
**T-stage-specific survival curves for malignant melanoma**. The predicted survival curves are based on the 50 completed data sets.

**Figure 3:**
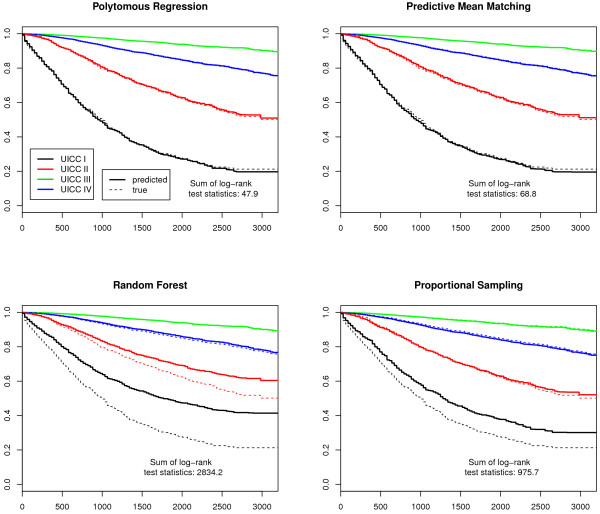
**UICC-stage-specific survival curves for female breast cancer**. The predicted survival curves are based on the 50 completed data sets.

**Figure 4:**
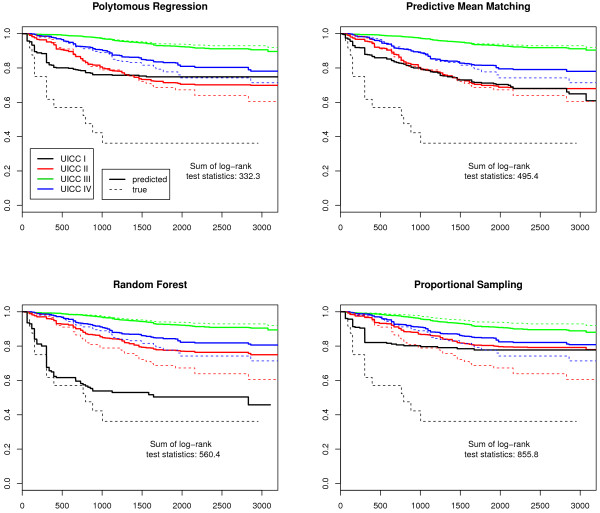
**UICC-stage-specific survival curves for malignant melanoma**. The predicted survival curves are based on the 50 completed data sets.

The sum of log-rank test statistics over all stages and imputations in Figures 1, 2, 3 and 4 indicate that the survival curves after multiple imputation with polytomous regression or predictive mean matching were closer to the observed survival curves than those after multiple imputation with random forests or proportional imputation. The log-rank statistics for UICC-stage for malignant melanoma were considerably higher than for T-stage or breast cancer (332.3 versus 80.8, 11.7 or 47.9 for polytomous regression).

### Sensitivity analyses

The results for malignant melanoma after multiple imputations with 25 imputations and 50 iterations altered only marginally and are not shown here. The greatest changes occurred for the random forest scenario, which can be explained by the large variance in all its estimations. Convergence of the multiple imputation algorithms was usually achieved very soon, i.e. even less than ten iterations would have sufficed. Only the random forest approach often did not converge at all.

## Discussion

Population-based cancer registry data is an important source for the evaluation of early detection programmes. Stage-specific analysis of incidence is especially crucial. A decrease in incidence of cancer with poor prognosis might be a strong indicator for future mortality reduction [1,2]. However, it is almost impossible to collect all data without missing information on the tumour stage, even if the cancer registry has complete registration. Missing data on tumour stage poses as a serious problem in the evaluation of early detection programmes. Completing the data set by an active follow-back, such as repeated record inspection, physician interview or other strategies is desirable, would, however, involve high costs and be very time consuming. Thus, appropriate alternatives in handling the unknown information in the cancer registry data set should be used. In this analysis, different variants of multiple imputation were studied, with respect to their feasibility and appropriateness for the imputation of missing values in tumour stages, limiting our analysis to one cancer entity with a high number of cases with missing tumour stage information and one cancer entity with only few missing tumour stage data.

### Multiple Imputation

A flexible and common approach of dealing with missing values is multiple imputation [15-17,30]. Multiple imputation with chained equations works as follows: for each variable with missing values an individual imputation model is fitted. The predictor variables are related to the missingness and/or to the value of the respective variable. The incomplete data set is completed by iterative imputation of the missing values with the corresponding imputation model. This is done *m *times, generating *m *completed data sets. Now the statistical analysis of interest is performed with each data set separately. Finally the *m *results are pooled to one result [31]. The application of multiple imputation is more complex than the use of other missing-data-approaches [32].

There are two main advantages of multiple imputation. First, in contrast to complete case analysis, all information in the data set is used in the analysis and the results are less likely to be biased. Second, missing values can only be imputed with some degree of uncertainty. In contrast to single imputation methods this uncertainty is reflected by the variability of the *m *results [33,34].

### Difference between T- and UICC-stage predictions

Only about 20% of the imputed values for UICC-stage are different from the observed values, while about 50% of the T-stage imputations are dislocated by at least one stage. It has to be taken into account that UICC-stage is generated from the three stage variables T, N and M and in many cases only one or two of them are missing.

Although the UICC-stage imputations for malignant melanoma correspond so well to the observed values on the individual level, the predictions of the stage-specific numbers of cases and survival curves were not accurate. This is due to the fact that the percentage of missing values is much higher in malignant melanoma cases and therefore a percentage of 50% dislocated imputed stage values has a greater impact in the total data set.

### Choice of the most appropriate imputation model

Machine learning techniques have been reported to produce better results than other classification models in situations with complex relations such as interactions or nonlinear relations [23,35]. Thus, we used random forests as an imputation model in addition to the two methods, which were already implemented in the mice-package in R (polytomous regression and predictive mean matching) to compare them to proportional sampling.

Overall, the imputation scenario based on polytomous regression seems to yield the best results. Imputed stage values are closest to the observed values; the difference of stage-specific numbers of cases to the observed data is smallest and the stage-specific survival curves fit best to the observed ones.

The predictive mean matching scenario yields results nearly as accurate as those by polytomous regression. It has the advantage of a shorter processing time and was found to be an appropriate method for imputation of missing values in other studies [36]. An explanation for the slightly better results of the polytomous regression might be a nonlinearity of the stages.

Using regression trees as imputation models for multiple imputation was found to be promising elsewhere [23]. Random forests, which consist of many regression trees, produce more stable results than a single regression tree. However, in the context of our study the estimations by the random forest scenario tended to have very large variances and were the most biased of all four scenarios. This might be due to convergence problems in the data completion; random forests are able to model complex relations, but if there is a lot of noise in the data, a random forest fits the model to this noise and the model fits can alter to a great extent from one iteration to the next. A simpler model such as polytomous regression or predictive mean matching seems to fit our data better.

The amount of missing values for the UICC-stage for malignant melanoma was too high to permit reasonable estimations by any of the applied methods. This agrees with other findings [36], that estimates are biased when the proportion of missing data exceeds 50%. In this case, the imputation can be strongly influenced by noise and produce biased results. In such a situation the proportional sampling approach, which does not depend on any covariates and cannot be influenced by their noise, yielded better estimations of the stage-specific numbers of cases. However, this approach makes the strong and probably inapplicable assumption that the stage distribution in the unknown stages is the same as in the observed stages. The conventional method of assigning all cases with unknown stage proportionally to the known stages - which is equivalent to proportional sampling with only one imputation - has the additional drawback of single imputation compared to multiple imputation.

### Strengths and limitations

The cancer registry in Schleswig-Holstein has a high completeness: it is estimated to be almost 100% [37]. Therefore, the only possible source for bias in the stage-specific numbers of case estimates is a biased imputation model in the multiple imputation procedure. The high completeness also reduces the risk of biased imputation models because no significantly different subgroups are missing in the model building.

A simulation study has the great advantage of making the results of the different methods comparable, because the true results are known.

One limitation of our small simulation study is the restriction to one scenario for the generation of the simulated data sets, that is the exclusion of the cases with missing T-, N- or M-stage from the simulated data sets. If these cases differ substantially from the other cases, the results of the analyses are not directly transferable. The same problem would occur if the real missingness-pattern differs substantially from the model we fitted from the original data set and used to generate the simulated data sets. The greatest difference between the simulated data set and the original data sets is the higher amount of missing values in UICC-stage. The values in T-, N- and M-stage were removed with separate models, which lessens the correlation between the missingness in these three variables. Thus, there were more cases to fill in, but the same number of missing values had to be imputed. Another difference occurred because a disproportionally high number of cases with a short survival time was omitted from the malignant melanoma data set. This is not seen as a bias in the data sets, because the shorter survival time probably only means a shorter registration time, i.e. less time to get notifications on T-, N- and M-stage. For the other variables, the univariate distributions in the simulated data set did not differ very much from those in the original data set.

The simulation of each five data sets for both cancer entities was aimed at controlling the variation of results due to the random deletion of values, but it is still a small number of simulations.

A proper simulation study would require additional scenarios in the design of simulated data sets, a higher number of simulations and a more detailed reporting of the results of the simulation study [20]. However, this limited simulation study appeared to be sufficient at identifying feasible imputation methods that provide reasonable results in cancer epidemiology.

Another limitation is that the imputation models do not take into account that the follow-up period for the recently diagnosed patients is quite short, which leads to very short survival times for patients who are alive and who may have a very good prognosis. We attempted to address this problem by including an interaction term for censoring and survival time and employing a random forest as imputation model, which might be capable of modelling such complex relations. The inclusion of year of diagnosis as predictor variable also helped to model this effect.

Further, the results are restricted to the data on two cancer entities of one cancer registry.

## Conclusions

For statistical analysis of tumour stage information in cancer registry data, both on the individual and the aggregated level, multiple imputation with chained equations using polytomous regression or predictive mean matching as an imputation model was in this limited simulation study found to be an appropriate method for dealing with missing data in tumour stage. Utilizing one of these methods should lead to less biased estimates than using a crude proportional method. Polytomous logistic regression and also predictive mean matching regression as imputation models for T-, N- and M-stage yield good estimations on the individual stage value, the stage-specific numbers of cases and the stage-specific survival curves, as long as the amount of missing values is not too high. In contrast, random forests are not recommended because convergence problems in the multiple imputation were observed, the results are less close to the observed parameters and have often large variances.

## Competing interests

The authors declare that they have no competing interests.

## Authors' contributions

NE made substantial contributions to the conception and design, the acquisition of the data, the statistical analysis and the interpretation of the data and was involved in drafting the manuscript. AW made substantial contributions to the analysis and the interpretation of the data and was involved in drafting the manuscript. AK made substantial contributions to the conception and design, the acquisition of the data, the statistical analysis and the interpretation of the data. All authors read and approved the final manuscript.

## Pre-publication history

The pre-publication history for this paper can be accessed here:

http://www.biomedcentral.com/1471-2288/11/129/prepub

## Supplementary Material

Additional file 1 **Definition of the TNM-categories**. Definition of the TNM-categories for malignant melanoma (ICD-10 C43) and breast cancer (ICD-10 C50) according to the TNM5- and the TNM6-classification. Click here for file

Additional file 2 **Definition of the UICC-stages**. Definition of the UICC-stages for malignant melanoma (ICD-10 C43) and breast cancer (ICD-10 C50) according to the TNM5- and the TNM6-classification. Click here for file
