# Supplementary material for: Imputation of missing values of tumour stage in population-based cancer registration
Source: BMC Med Res Methodol. 2011 Sep 19;11:129. doi: 10.1186/1471-2288-11-129 (PMC3184281; doi:10.1186/1471-2288-11-129)
Supplement: Additional file 1 — Definition of the TNM-categories. Definition of the TNM-categories for malignant melanoma (ICD-10 C43) and breast cancer (ICD-10 C50) according to the TNM5- and the TNM6-classification. [file 1471-2288-11-129-S1.PDF]

	Breast cancer (C50)		Melanoma (C43)	
	**TNM5**	**TNM6**	**TNM5**	**TNM6**
**T-stage**				
1	Greatest dimension $\leq$ 2cm		Thickness $\leq$ 0.75 mm and invades the papillary dermis	Thickness $\leq$ 1 mm
2	2 – 5cm		0.75-1.5mm or invades to the papillary-reticular dermal interface	1-2mm
3	> 5 cm		1.5-4mm or invades the reticular dermis	2-4mm
4	Invades adjacent tissue		>4mm or invades the subcutaneous tissue or satellite within 2cm of the primary tumour	> 4mm
X	Cannot be assessed		Cannot be assessed	Cannot be assessed
**N-stage**				
0	No regional lymph node metastasis	No regional lymph node metastasis	No regional lymph node metastasis	No regional lymph node metastasis
1	Metastasis to movable ipsilateral axillary lymph node(s)	Metastasis to movable ipsilateral axillary lymph node(s)	Metastasis $\leq$ 3cm in greatest dimension in any regional lymph node(s)	1 regional lymph node metastasis
2	Metastasis to ipsilateral axillary lymph node(s) fixed to one another or to other structures	Metastasis to ipsilateral axillary lymph node(s) fixed to one another or to other structures,<br>or to clinically apparent ipsilateral internal mammary lymph nodes without axillary nodes	Metastasis >3cm in greatest dimension in any regional lymph node(s) or in-transit metastasis	2-3 regional lymph node metastases or satellite or in-transit metastasis without metastasis in lymph node(s)
3	Metastasen to ipsilateral internal mammary lymph node(s)	Metastasis to ipsilateral infraclavicular lymph node(s) with or without axillary lymph node(s),<br>or to clinically apparent ipsilateral internal mammary lymph nodes<br>with axillary nodes,<br>or metastasis to ipsilateral supraclavicular lymph node(s) with or without axillary lymph nodes or internal mammary lymph node(s)	-	> 4 lymph node metastasis, or matted metastatic regional lymph nodes, or satellite or in-transit metastasis with metastasis in regional lymph node(s)
X	Cannot be assessed	Cannot be assessed	Cannot be assessed	Cannot be assessed
**M-stage**				
0	No distant metastasis		No distant metastasis	
1	Distant metastasis		Distant metastasis	
X	Cannot be assessed		Cannot be assessed	
