# Supplementary material for: Imputation of missing values of tumour stage in population-based cancer registration
Source: BMC Med Res Methodol. 2011 Sep 19;11:129. doi: 10.1186/1471-2288-11-129 (PMC3184281; doi:10.1186/1471-2288-11-129)
Supplement: Additional file 2 — Definition of the UICC-stages. Definition of the UICC-stages for malignant melanoma (ICD-10 C43) and breast cancer (ICD-10 C50) according to the TNM5- and the TNM6-classification. [file 1471-2288-11-129-S2.PDF]

UICC-stage	Melanoma						Breast cancer		
	TNM 5			TNM 6			TNM5 and TNM6		
I	T1, T2	N0	M0	T1, T2a	N0	M0	T1	N0	M0
II	T3	N0	M0	T2b, T3, T4	N0	M0	T2, T3	N0	M0
							T1, T2	N1	M0
III	T4	N0	M0	Any	N1, N2, N3	M0	T3	N1	M0
	Any	N1, N2	M0				T4	Any	M0
							Any	N2, N3	M0
IV	Any	Any	M1	Any	Any	M1	Any	Any	M1
